# Systematic Literature Review on Public Health Impacts of Persistent Tic Disorders: Education and Employment

**DOI:** 10.1007/s10567-025-00537-3

**Published:** 2026-01-03

**Authors:** Helena J. Hutchins, Patricia Whalen, Jorge Verlenden, Hidayat Ogunsola, Brooke S. Staley, Rebecca T. Leeb, Wendy Wegman, Rebecca H. Bitsko

**Affiliations:** 1 Applied Research and Evaluation Team, Division of Human Development and Disability, National Center On Birth Defects and Developmental Disabilities, Centers for Disease Control and Prevention, 4770 Buford Hwy S106-4, Atlanta, GA 30341-3717 USA; 2 Oak Ridge Institute for Science and Education, CDC Research Participation Programs, Oak Ridge, TN USA; 3 Division of Adolescent and School Health, National Center for Chronic Disease Prevention and Health Promotion, Centers for Disease Control and Prevention, 4770 Buford Hwy, Atlanta, GA USA; 4 Epidemic Intelligence Service, CDC, Atlanta, GA USA; 5 Tourette Association of America, 42-40 Bell Boulevard, Suite 507, Bayside, NY 11361 USA

**Keywords:** Tourette syndrome, Tic disorder, Education outcomes, Employment

## Abstract

Tourette syndrome and persistent tic disorders (TS/PTD) begin in childhood and can contribute to negative outcomes across the lifespan. A systematic review was conducted to summarize current evidence on education and employment outcomes among individuals with TS/PTD. The review summarized education and/or employment outcomes from 69 articles published between 2003 and March 5, 2025 that reported these outcomes for individuals with TS/PTD and a comparison group without TS/PTD. Of these studies, most included small samples of individuals with TS/PTD (less than 100), and those that reported on race or ethnicity were predominantly White. Only five studies on adult employment status and no studies on the transition to higher education were identified for inclusion. Children and adolescents with TS/PTD may experience poorer school-related quality of life, lower school competence, and more parent-reported school problems compared to those without TS/PTD. Although evidence was less robust, children and adolescents with TS/PTD may also be more likely to experience other negative school outcomes, including poor attitudes about school and low pass rates/frequent grade retention. Findings can be used by healthcare providers and school personnel to inform supports for students with TS/PTD. Adult employment status and transition to higher education for individuals with TS/PTD, and studies with larger, more heterogeneous samples, may be important directions for future research.

**Supplementary Information:**

The online version contains supplementary material available at 10.1007/s10567-025-00537-3.

## Introduction

Tourette syndrome and persistent tic disorders (TS/PTD) typically have an onset between 4 and 6 years of age, and are characterized by the presence of motor or vocal tics which persist for at least one year (American Psychiatric Association, 2013, 2022). Boys are 2–4 times more likely to have a TS diagnosis compared to girls (American Psychiatric Association, 2013, 2022; Claussen et al., 2018). Tics vary in severity, frequency, and complexity and can persist into adulthood (American Psychiatric Association, 2013, 2022; Black et al., 2021; Leckman et al., 1989). Approximately 80% of individuals with TS/PTD have at least one co-occurring disorder (e.g., attention-deficit/hyperactivity disorder (ADHD), obsessive–compulsive disorder (OCD), learning disorders) (American Psychiatric Association, 2013, 2022; Black et al., 2021; Claussen et al., 2018; Gorman et al., 2010).

Symptoms of TS/PTD and co-occurring disorders can contribute to a range of negative outcomes across the lifespan, including those related to education and employment (American Psychiatric Association, 2013; Claussen et al., 2018; Ricketts et al., 2022a; Yang et al., 2016). TS/PTD are not associated with lower intelligence (Channon et al., 2009; Gadow et al., 2009; Lin et al., 2012; Lund et al., 2023) suggesting that education outcomes for people with TS/PTD would be analogous to those of people without tic disorders. However, because tics may interfere with the learning process directly (e.g., impacting handwriting) or indirectly (e.g., fatigue resulting from attempts to suppress tics at school) students with TS/PTD may need supports at school, including special education (Mingbunjerdsuk & Zinner, 2020). Furthermore, tics can draw unwanted attention, resulting in stigma, social isolation, and bullying; attempts by school staff to manage tics may lead to harmful discipline practices such as exclusion, suspension, and expulsion (Zinner et al., 2012).

Education is an important social determinant of health (Wong et al., 2022). Lower levels of education, including dropout prior to high school completion, are associated with poor health, decreased healthcare access, and higher disease mortality (Balaj et al., 2024; Zajacova & Lawrence, 2018). Educational attainment is associated with future employment status and earnings, which are also associated with health outcomes (Hergenrather et al., 2015; U.S. Bureau of Labor Statistics, 2020).

Knowledge of education and employment outcomes associated with TS/PTD can inform efforts to prevent and mitigate school challenges and support individuals with these disorders. This systematic review aims to synthesize indicators of education and employment outcomes associated with TS/PTD (herein referred to as outcomes).

## Methods

We conducted a broad literature search to identify outcomes associated with TS/PTD; the full search methodology is described elsewhere (Bitsko et al., 2025) and summarized here. This paper synthesizes literature on education and employment. Other outcome categories of interest included in our broad review, but not included in this paper, are healthcare (Bitsko et al., 2025), physical health, health risk behaviors, social and family outcomes, and quality of life.

The original literature search, conducted on July 6, 2023, using multiple databases (Medline, Embase, PsycInfo, Cochrane library, CINAHL, and Scopus) identified 6510 articles published in English. Two reviewers screened the title and abstract of each article in Covidence; a third reviewer adjudicated disagreements. We excluded 4076 articles with any of the following characteristics: (1) involved non-human subjects, (2) included research protocol only, (3) focused on non-relevant “tics” (e.g., trauma informed care [TIC]), (4) focused on tardive dyskinesia, Parkinson’s disease, schizophrenia, Huntington’s disease, dystonias, functional tics, psychogenic tics, transient tics, or provisional tics rather than TS/PTD, (5) was a book review, video, poster, slide, or personal account, (6) was not peer reviewed, (7) reported on a study with less than 10 participants, or (8) was a conference abstract.

**Fig. 1:**
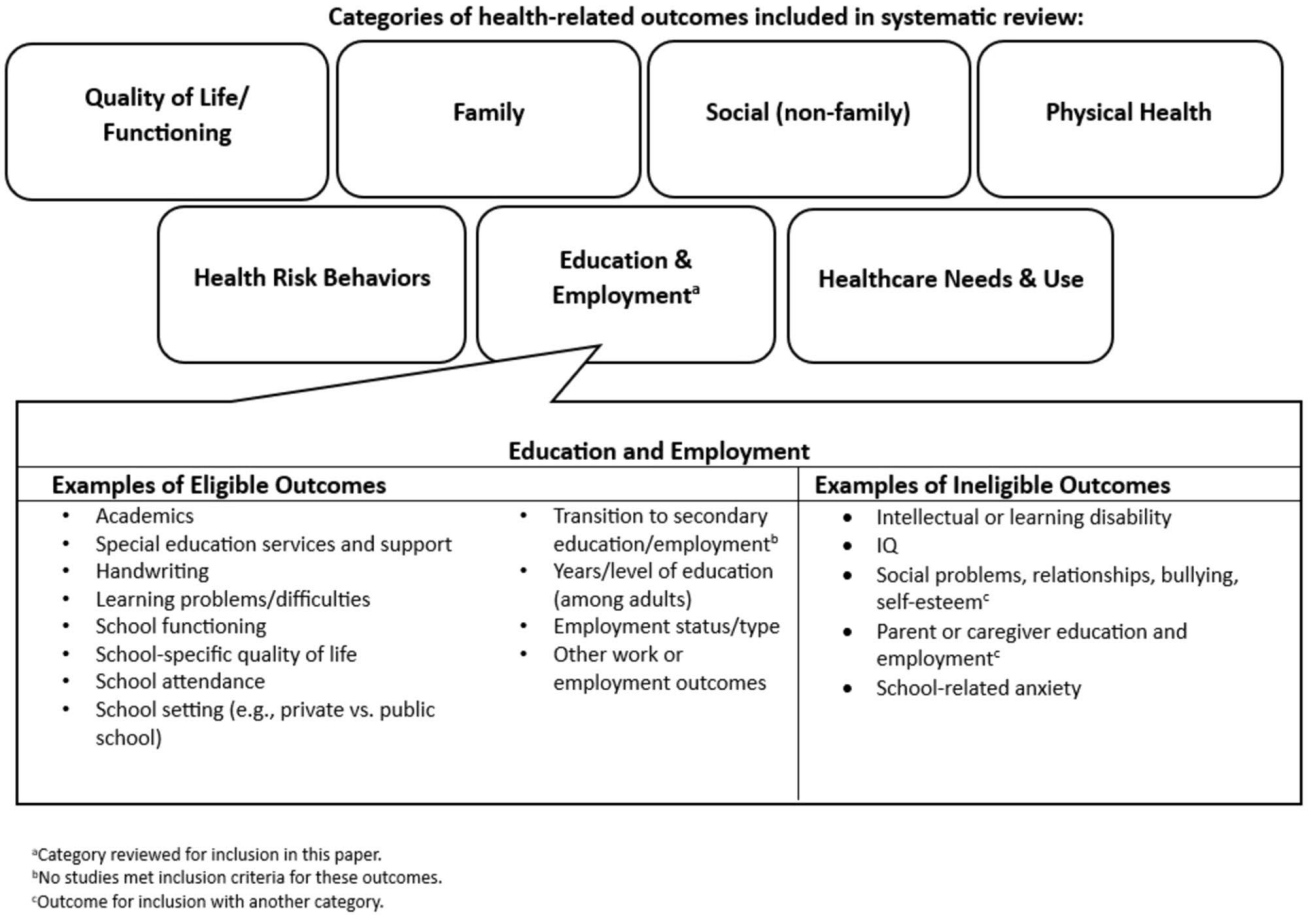
Overarching categories of health-related outcomes included in the systematic review and examples of eligible and ineligible outcomes within the education and employment category. **a** Category reviewed for inclusion in this paper. **b** No studies met inclusion criteria for these outcomes. **c** Outcome for inclusion with another category

Full-text screening of the remaining 2434 articles was conducted by two reviewers; if exclusion criteria were clearly present, a second reviewer was not required. Eligible outcomes were those identified as potentially modifiable through public health activities (e.g., academic performance and learning problems were included but learning disability and IQ were excluded; Fig. 1). Overarching categories and examples of eligible and ineligible outcomes were identified based on existing knowledge and refined throughout the review to reflect available evidence; changes were retrospectively applied to previously screened articles. Additional exclusion criteria during full text screening included: (1) articles with no eligible outcomes (see below and Fig. 1), (2) reviews and meta-analyses, and (3) surveys of healthcare providers or educators about TS/PTD. This resulted in the exclusion of 1826 articles; five additional articles were identified and screened during full text review by reviewing cited methodology papers.

Of 613 articles remaining, two reviewers extracted those published in 2003 or later that reported outcomes for individuals with TS/PTD and a comparison group without TS/PTD (*n* = 173) in Covidence; a third reviewer conducted a consensus review. The extracted data are summarized in Tables 1, 2, 3, 4, 5, 6, 7, 8, 9, 10, and 11. We used the Newcastle–Ottawa Scale to assess quality, sample selection, and comparability in cohort and case/control studies, assessment of outcome in cohort studies, and ascertainment of exposure (i.e., tic disorder status) in case–control studies (Luchini et al., 2017; Margulis et al., 2014; Moskalewicz & Oremus, 2020).

**Table 1:** Characteristics of studies included in *Systematic Literature Review on Public Health Impacts of Persistent Tic Disorders: Education and Employment*

Author, Year	PTDs included (n)	Mode of assessment; PTD criteria	Comparison population (n)	Age at outcome (years)	Male (%)	Race/ethnicity (% or n)	Sample type for cases; CG *if distinct*	Years of data collection (country)	Outcome categories included
Balottin et al., 2016	Current TS (17)	Previously diagnosed; DSM-IV-TR	Adolescents/young adults with no neuropsychiatric history (51)	12–19	TS: 100% CG: 100%	NR	TS specialty clinic; local schools	2006–2013 (Italy)	School competence; School problems
Berg et al., 2024a	Current TS (22)	Previously diagnosed; NR	Ch/adol/young adults with functional tic-like behaviors (35)	11–25	TS: 9.1% CG: 8.6%	Ethnic minority TS: 27.3% FTLB: 21.2%	Movement disorders clinic	2021–2022 (Canada)	School absences; Employment
Berg et al., 2024b	Current TS (22)	Clinical assessment; DSM-5	Ch/adol/young adults Neurotypical (25)	11–25	TS: 9.1% CG: 12.0%	NR	Movement disorders clinic; recruitment posters from research program	2020–2022 (Canada)	Academic performance; Employment
Chalita et al., 2012	Current symptoms suggestive of tic disorder (NR)	Self-report by child; NR	nc, adolescents (NR) [total sample including PTD = 237]	12–15	38.4% of total sample	"No significant racial-ethnic diversity"	School-based	nd (Mexico)	School type and student status
Channon et al., 2003	Current TS (21)	Clinical assessment; NHIS-TS, DSM-IV-TR	nc, adults “healthy controls” (21)	18–65	TS: 85.7% CG: 81.0%	NR	NR	nd (U.K.)	Years of education
Channon et al., 2006	Current TS (20)	Previously diagnosed, clinical assessment; NHIS-TS, DSM-IV	nc, adults “healthy controls” (25)	18–50	TS: 65.0% CG: 64.0%	NR	NR	nd (U.K.)	Years of education
Channon et al., 2009	Current TS (21)	Previously diagnosed, clinical assessment; NHIS-TS, DSM-IV-TR	Adults without TS (23)	18–60	TS: 57.1% CG: 60.9%	NR	TS specialty clinic; university research participant pool	nd (U.K.)	Years of education
Claussen et al. 2018	Current TS (300)	Previously diagnosed, parent-report; NSCH	Ch/adol with no history of TS (129,053)	6–17	TS: 81.8% CG: 51.1%	TS: 84.9% W, 4.4% B, 10.7% MR/O, 10.2% H CG: 69.2% W, 16.3% B, 14.6% MR/O, 20.8% H	National survey (NSCH)	2007–2008 & 2011–2012 (U.S.)	Attitudes about school; School absences; school type and student status; Special education services and support; Pass rate and grade retention; Academic performance; School problems
Cloes et al. 2017	Current TS (85)	Previously diagnosed, clinical assessment; DSM-IV-TR	Ch/adol w/o psychiatric, neurological, developmental, or major medical disorders (92)	9–17	TS: 84% CG: 48%	TS: 97% W, 1% B, 1% H CG: 78% W, 9% B, 2% A, 10% MR	TS specialty clinic; hospital marketing service	2011–2013 (U.S.)	School impairment and limitations
Colautti et al. 2023	Current TS (25)	Clinical assessment; DSM-5	Adult healthy controls (25)	Mean TS: 25.9 CG: 26.5	TS: 76.0% CG: 76.0%	NR	TS specialty clinic; referred by TS participants	2021–2022 (Italy)	Employment
Cubo et al. 2013	Current TD (n = 162, 28% with impairment criteria and 72% without impairment criteria)	Clinical assessment; DSM-IV-TR	Ch/adol without current tics or TDs (245)	6–16	TD: 68.5% CG: 60.7%	TS: 92.5% W CG: 88.4% W	School-based	2007–2009 (Spain)	Academic performance; learning difficulties; Special education services and support; School type and student status; School absences
Cubo et al. 2017	Current TD or nonspecific tics (61)	Clinical assessment at baseline and follow-up; DSM-IV-TR	Ch/Adol without current or history of tic disorder (156)	11–17	TD: 63.9% CG: 52.6%	NR	School-based: students w/o any history of grade retention, with and without TD, identified during the first wave of the study (Cubo 2013) who were still at school	2010–2014 (Spain)	Pass rate and grade retention; Special education services and support; School type and student status
Cutler et al. 2009	Current TS (57)	Previously diagnosed, clinical assessment; MOVES	Ch/adol normative sample, (1,033)	TS: 8–17 CG: 8–18	TS: 80.7% CG: NR	TS: 96% W, 4% MR (of 25 with data on race) CG: NR	TS specialty clinic; normative sample from previous research	2004–2007 (U.K.)	School-related quality of life
Debes et al. 2010	Current TS (314)	Previously diagnosed, clinical assessment; DSM-IV-TR	Ch/adol without tics or any psychiatric disorders or chronic somatic diseases (81)	Ch/Adol > 10	TS: 80.7% CG: 65.4%	NR	TS specialty clinic; local schools	2005 (Denmark)	School type and student status
Deckersbach et al. 2006	Current TS (35)	Clinical assessment; DSM-IV	Adults w/o TS (20)	Mean TS: 35.1 CG: 34.3	TS: 62.9% CG: 60%	NR	OCD clinic; hospital advertising	nd (U.S.)	Years of education
Drury et al. 2016	Current TS (20)	Previously diagnosed, clinical assessment; DSM-IV-TR	Adults w/o TS (20)	TS: 18–60 CG: 18–60	TS: 75% CG: 75%	NR	TS specialty clinic; participant recruitment website	nd (U.K.)	Education level; Years of education
Eddy et al. 2010a	Current TS (18)	Previously diagnosed, clinical assessment; DSM-III-R, DSM-IV-TR, NHIS-TS	nc, adolescent/adult “healthy controls” (10)	TS: 17–54 CG: 17–41	TS: 50% CG: 70%	NR	TS specialty clinic; NR	nd (U.K.)	Years of education
Eddy et al. 2010b	Current TS (16)	Previously diagnosed, clinical assessment; DSM-III-R, DSM-IV-TR, NHIS-TS	nc, adult “healthy controls” (8)	Mean TS: 32.1 CG: 34.3	TS: 81.3% CG: 62.5%	NR	TS specialty clinic; NR	nd (U.K.)	Years of education
Eddy et al. 2011	Current TS (18)	Previously diagnosed, clinical assessment; DSM-III-R, DSM-IV-TR, NHIS-TS	nc, adolescent/adult “healthy controls” (20)	TS: 16–47 CG: 18–37	TS: 72.2% CG: 45%	NR	TS specialty clinic; NR	nd (U.K.)	Years of education
Eddy et al. 2012	Current TS (40)	Previously diagnosed, clinical assessment; DSM-IV-TR, NHIS-TS	nc, adolescent/adult “healthy controls” (20)	TS: 16–64 CG: 18–55	TS: 72.5% CG: 75%	NR	NR	nd (U.K.)	Years of education
Eddy et al. 2014	Current TS (18)	Previously diagnosed, clinical assessment; DSM-III-R, DSM-IV-TR, NHIS-TS	nc, adolescent/adult “neurologically intact” controls (18)	TS: 16–61 CG: 18–61	TS: 72.2% CG: 50%	NR	NR; community/school staff and students	nd (U.K.)	Years of education
Eddy & Cavanna, 2015	Current TS (20)	Previously diagnosed, clinical assessment; DSM-V, NHIS-TS	Adults w/o TS or any psychiatric or neurological diagnosis (20)	TS: 19–68 CG: 18–65	TS: 85% CG: 85%	NR	NR	nd (U.K.)	Years of education
Erbilgin Gun & Kilincaslan, 2019	Current TD + ADHD (54)	Clinical assessment; DSM-IV-TR	Ch/adol/young adults with ADHD only (54)	7–18	TD + ADHD: 83.3% CG: 72.2%	NR	Child and adolescent psychiatric outpatient clinic	2014–2016 (Turkey)	School-related quality of life
Ezpeleta & Toro, 2009	TD (28)	Previously diagnosed, clinical assessment; DSM-IV	Ch/adol with ADHD only (131)	8–17	Total: 54.0%	Total: 97% W	Psychiatry outpatient clinics	nd (Spain)	School impairment and limitations
Fan et al. 2018	Current TS (23)	Previously diagnosed, clinical assessment; DSM-IV-TR	nc, “previously examined healthy controls” (22)	Mean TS: 34.6 CG: 41.4	TS: 65% CG: 60%	NR	Outpatient clinics and patient support organization and outpatient clinics; community-based	nd (Netherlands)	Years of education
Gadow et al. 2009	Current CMTD + ADHD (66, including 62 TS & 4 CMTD)	Clinical assessment; NR	Children with ADHD only (66)	Mean CMTD + ADHD: 8.6 CG: 8.0	CMTD + ADHD: 77% CG: 89%	CMTD + ADHD: 88% W, 12% O CG: 89% W, 11% O	Psychiatry and community clinics, schools, media, parent-support groups	nd (U.S.)	Special education services and support
Gomes de Alvarenga et al. 2012	Current OCD + TD (236)	Clinical assessment; SCID-I; DSM-IV	Adults with OCD only (557)	Mean TD + OCD: 32.4 CG: 35.9	TD + OCD: 49.2% CG: 38.5%	TD + OCD: 84.7% W, 3.0% Afro-Brazilian, 0.8% A, 11.0% MR, 0.4% O CG: 82.1% W, 4.7% Afro-Brazilian, 1.4% A, 11.6% MR, 0.2% O	Outpatient clinics (Brazilian OCD Research Consortium)	2003–2008 (Brazil)	Education level
Gorman et al. 2010	Current TS (65)	Previously diagnosed, clinical assessment; DSM-IV	Adolescents/young adults w/o any current or history of tics or OCS (65)	Mean TS: 18.1 CG: 18.4	TS: 87.7% CG: 87.7%	TS: 98.5% W, 1.5% NW CG: 98.5% W, 1.5% NW	TS (and OCD) specialty clinic; community-based	1993–1999 (U.S.)	School competence
Gutierrez-Colina et al. 2015	Current TS (39)	Previously diagnosed, parent-report; TODS-PR	Ch/adol normative sample (NR)	8–18	TS: 76.9% CG: NR	TS: 94.9% W, 5.1% B CG: NR	Specialty summer camp attendees; normative sample from previous research	nd (U.S.)	School-related quality of life
Guttmann-Steinmetz et al. 2009	Current CMTD + ADHD (47)	Clinical assessment; NR	Community-based controls (173)	Mean CMTD + ADHD: 8.6 CG: 8.1	CMTD + ADHD: 100% CG: 100%	CMTD + ADHD: 7% NW CG: 2% NW	Outpatient clinic; schools	nd (U.S.)	School absences
Guttmann-Steinmetz et al. 2010	Current CMTD + ADHD (51)	Clinical assessment; NR, “described in previous publication”	Community-based controls (170)	Mean CMTD + ADHD: 8.6 CG: 9.0	CMTD + ADHD: 100% CG: 100%	CMTD: 15% NW CG: 6% NW	Outpatient clinic; parents	nd (nc, includes U.S.)	Attitudes about school
Hao et al. 2010	Current TS (424)	Previously diagnosed, self-report; NR	Children w/o any acute or chronic diseases (1,583)	TS: 8–12 CG: 5–12	TS: 69.1% CG: 48.4%	NR	Clinic-based; school-based	nd (China)	School-related quality of life
Hesapcioglu et al. 2014	Current TS/CTD (57)	Previously diagnosed, clinical assessment; DSM-IV-TR	Ch/adol patients without TS/CTD (57)	6–16	NR	NR	Ch/adol psychiatry outpatient clinic	nd (Turkey)	School-related quality of life
Horesh et al. 2018	Current TS (132)	Previously diagnosed, clinical assessment; DSM-5	Ch/adol/young adult w/o TS (49)	8–18	TS: 78.0% CG: 65.3%	NR	TS specialty clinic; community-based	nd (Israel)	Academic performance
Jalenques et al., 2017	Current TS (75)	Previously diagnosed, clinical assessment; DSM-IV-TR	Adolescents/young adults without TS (75)	12–18	TS: 80.0% CG: 80.0%	NR	Clinic-based; community-based	2010–2013 (France)	School problems; Academic performance; Pass rate/grade retention
Jiang et al., 2025	Current severe TD + ADHD (1,140)	Clinical assessment; DSM-5	Current severe ADHD (2,317)	5–17	Severe TD + ADHD: 80.4% CG: 76.0%	NR	Clinic-based	2022–2023 (China)	Learning difficulties
Keenan et al., 2024	Current TS (12)	Previously diagnosed, clinical assessment; ICD-10	Children without tics or diagnosis of developmental, psychological, or sleep disorders (22)	8–12	TS: 70.0% CG: 59.1%	NR	Clinic- and community, patient support organizations; community	2021–2022 (Ireland)	School competence
Khalifa & Knorring, 2005	Current TS/CMT/CVT (25 TS + 34 CMT + 24 CVT)	Clinical assessment; DSM-IV	Ch/adol with transient tics (25)	7–15	NR	NR	Community-based	nd (Sweden)	Learning difficulties
Khalifa & Knorring, 2006	Current TS/CMT/CVT (25 TS + 34 CMT + 24 CVT)	Clinical assessment; DSM-IV	Ch/adol without tics (25)	7–15	nc, (CMT/CVT group: 40 boys) CG: NR	NR	School-based	1999–2003 (Sweden)	Special education services and support
Kurvits et al., 2024	Current TD (29)	Previously diagnosed, clinical assessment; DSM-5	Healthy controls (29)	Mean TD: 28.5 CG: 28.6	TD: 79% CG: 70%	NR	Neurology clinic; NR	nd (Germany)	Years of education
Lanzi et al., 2004	Current unspecified TD (68)	Teacher report; NR	Total study population including TDs (2,347)	TD: 6–11 Total: 5–12	TD: 82% Total: 54%	NR	School-based	1995–1996 (Italy)	Academic performance
Lavoie et al., 2007	Current TS/CTD (18 TS, 18 CTD)	Self-report, clinical assessment; DSM-IV-TR	Adults w/o CTD (22)	Mean CTD: 41 CG: 33	TS/CTD: 50% CG: 45.5%	NR	Community-based	nd (Canada)	Years of education
Lin et al., 2012	Current ADHD + TD, including TS, CTD, transient tic disorder (40)	Previously diagnosed; NR	Ch/adol without TD or ADHD (40)	8–16	ADHD + TD: 95% CG: 95%	NR	Child neurology/psychiatry clinics; school-based	nd (Taiwan)	Attitudes about school; School problems; School-related quality of life
Liu et al., 2017	Current TS (107)	Previously diagnosed, clinical assessment; DSM-IV-TR	Children with no psychiatric disorders (107)	Mean TS: 10.1 CG: 9.9	TS: 86% CG: 55.1%	NR	Clinic-based	2008–2011 (China)	School-related quality of life
Lund et al., 2023	Current TS only, TS + OCD, TS + ADHD (223, includes 93 with TS only)	Previously diagnosed, clinical assessment; DSM-IV-TR	Young adults with no psychiatric disorders (53)	Mean TS groups: 18.5 CG: 18.6	TS groups: 80.65% CG: 60.38%	NR	TS specialty clinic; school-based	2005–2007, 2011–2013 (Denmark)	Pass rate and grade retention
Moretto et al., 2011	Current TS (13)	Previously diagnosed, clinical assessment; DSM-IV	Adults w/o motor and psychological disorders (13)	TS: 18–65 CG: 21–58	TS: 84.6% CG: 84.6%	NR	TS specialty clinic; university volunteer subject pool	nd (U.K.)	Years of education
Muller et al., 2003	Current TS + OCD (14)	Clinical assessment; DSM-IV	Adults w/o neurological or psychiatric disorders (14)	18–62	TS + OCD: 92.9% CG: 92.9%	NR	NR	nd (Germany)	Years of education
Muller-Vahl et al., 2020	Current TS/CTD (115 TS + 12 CTD)	Previously diagnosed; NR	Adults w/o CTD (645)	≥ 18	CTD: 70.1% CG: 20.6%	NR	TS specialty clinic and patient support organizations; school-based staff and students	nd (Germany)	Education level
Neuner et al., 2010	Current TS (19)	Previously diagnosed, clinical assessment; DSM-IV-TR	Adults w/o neurologic/psychiatric disorders (19)	Mean TS: 29.7 CG: 29.7	TS: 73.7% CG: 73.7%	NR	Clinic-based; employees within the research center and university students	nd (Germany)	Years of education
O’Connor et al., 2014	Current PTD as primary presenting problem (18 TS only + 42 CTD)	Previously diagnosed, clinical assessment; DSM-IV/SCID-I-P	Adults with tics with BFRBs as the primary presenting problem (36)	18–65	nc	NR	OCD clinic	nd (Canada)	Education level
O’Hare et al., 2016	Current TS (86)	Previously diagnosed; NR	Ch/adol with no known psychiatric or medical diagnoses (108)	7–16	TS: 85.4% CG: 73.1%	TS: 87.1% W, 2.4% A/TSI, 3.5% A, 7.1% O CG: 99.1%W, 0.9% O	Patient support organizations; nation-wide advertisements	nd (Australia)	School-related quality of life
Palminteri et al., 2009	Current TS, not taking medication (12)	Previously diagnosed; NR	Adolescents/young adults w/o any history of neurological or psychiatric conditions (24)	Mean TS: 21.3 CG: 22.3	TS: 75% CG: 50%	NR	TS specialty clinic; NR	nd (France)	Years of education
Perez-Vigil et al., 2018	Current TS/CTD (3,590)	Previously diagnosed; ICD-8, ICD-9, ICD-10	NSR non-PTD study population (2,111,964)	Born in Sweden 12/1/1976–12/31/1998, w/available info until 12/31/2013	TS/CTD: 78.6% CG: 51%	NR	Registry (NSR)	1976–2013 (Sweden)	Education level; Pass rate and grade retention
Poh et al., 2018	Current CTD + ADHD (23)	Clinical assessment; DISC-IV, DSM-IV	Children with ADHD only (92)	7–10	NR	NR	School-based	2011–2015 (Australia)	Academic performance; School-related quality of life
Pringsheim et al., 2009	Current TS (71)	Clinical assessment; DSM-IV-TR	Ch/adol without TS (391)	7–17	TS: 78.9% CG: NR	NR	TS specialty clinic; normative data	2006–2007 (U.S. and Canada)	School impairment and limitations
Rae et al., 2018	Current TS (21)	Previously diagnosed, clinical assessment; NR	Adults with no history of any major neurological or psychiatric disorder (21)	TS: 18–51 CG: 19–55	TS: 61.9% CG: 52.4%	NR	Neurobehavioral clinic and patient support organization; NR	nd (U.K.)	Years of education
Ricketts et al., 2022a	Current TS only (30)	Previously diagnosed, parent-report; NS-DATA	Ch/adol with ADHD only (2,899)	4–17	TS: 86.2% CG: 69.9%	TS: 10.4% M CG: 37.6% M	National survey (NS-DATA)	2014 (U.S.)	Academic performance
Ricketts et al., 2022b	Current TD (14)	Previously diagnosed; DSM-5	Adults w/o current or history of DSM-5 psychiatric disorders (20)	22–50	TD: 71.4% CG: 65%	TD: 21.4% M CG: 60% M	Academic medical center	nd (U.S.)	Education level; Employment
Salvador et al., 2017	Current TS (17 unmedicated for TS, 14 doing aripiprazole monotherapy for TS)	Previously diagnosed, clinical assessment; DSM-5	nc, adult “healthy controls” (20)	> 18	TS: 71% CG: 55%	NR	TS specialty clinic; advertisements	nd (France)	Years of education
Storch et al., 2007	Current TS/CTD (59)	Previously diagnosed, clinical assessment; DSM-IV-TR	nc, ch/adol “healthy controls” (386–401 depending on outcome)	8–17	TS/CTD: 69.5% CG: NR	TS/CTD: 97% W, 3% H CG: NR	TS specialty clinic; control sample data from previous publications	nd (U.S.)	School-related quality of life
Termine et al., 2006	Current TS (17)	Previously diagnosed, clinical assessment; DSM-IV	Ch/adol with no current or history of TS (17)	TS: 7.3–17.4 CG: 7.1–17.3	TS: 82.4% CG: 82.4%	NR	Child neurology/psychiatry clinic; school-based	2000–2002 (Italy)	School competence
Termine et al., 2022	Current TD (49)	Previously diagnosed, clinical assessment; DSM-5	Ch/adol without TDs (245)	TS: 6–18 CG: 2–18	TS: 79.6% CG: 79.6%	NR	Child and Adolescent Psychiatry clinic and patient support organization	2020 (Italy)	Learning difficulties
Warren et al., 2020	Current TS (23)	Previously diagnosed, clinical assessment; DSM-IV-TR	Adults with no current or history of any significant neurological or psychiatric conditions (27)	TS: 18–58 CG: 19–49	TS: 56.5% CG: 59.3%	NR	TS specialty clinic; community-based	2014–2015 (Germany)	Years of education
Watson et al., 2024	Current TS (38)	Previously diagnosed, clinical assessment; DSM-5	Adolescents without tics (28)	13–17	TS: 63.2% CG: 50.0%	TS: 86.8% W, 0% A, 2.6% B, 7.9% MR, 2.6% NH/PI, 7.9%, H, 92.1% NH CG: 78.6% W, 3.6% A, 7.1% B, 10.7% MR, 0% NH/PI, 3.6% H, 96.4% NH	TS specialty clinic; Word of mouth, Pediatric clinics, Vanderbilt Research list	2021–2022 (U.S.)	Learning difficulties
Wei, 2011	Current TS (127)	Previously diagnosed; DSM-IV	“Typically developing” children (138)	6–12	TS: 83.5% CG: 83.3%	NR	School-based	nd (Taiwan)	Academic performance; Attitudes about school
Worbe et al., 2011	Current TS (60)	Previously diagnosed; NR	Adults w/o any history of neurologic or psychiatric disorders (50)	Mean TS: 30.1 CG: 27.1	TS: 68.3% CG: 54.0%	NR	TS specialty clinic; NR	nd (France)	Education level
Yang et al., 2016	Current TS (122)	Previously diagnosed, self-report; CCHS	Adolescent/adult survey participants w/o TS (122,884, including those with TS)	> 12	TS: 74.7% CG: 49.3%	NR	National survey (CCHS)	2010–2011 (Canada)	Education level; Employment
Zhu et al., 2006	Current TS (69)	Previously diagnosed, clinical assessment; DSM-IV	Ch/adol w/o any psychiatric, gross neurological, or other organic disorders (69)	8–16	TS: 84.1% CG: 84.1%	NR	Mental health outpatient clinic; school-based	2000–2002 (China)	School competence
Zinna et al., 2021	Current TS + CTD (43)	Previously diagnosed, clinical assessment; ICD-10	Children w/o TS/CTDs (175)	≤ 13	TS/CTD: 67.5% CG: 50.9%	NR	Psychiatry/neurology/psychology clinics	2009–2019 (U.K.)	School type and student status

Age Categories: Child =  < 12 years, Adolescent = 12–17 years, Ch/adol =  < 18 years, Young Adults = for sample groups that are 18–25 years old, Adult = 18 + years

Race/ethnicity: A = Asian, A/TSI = Aboriginal or Torres Straight Islander, B = Black, H = Hispanic, M = Minority, MR = Multiple Races, NA/AN = Native American or Alaskan Native, NH/PI = Native Hawaiian or other Pacific Islander, NH = Non-Hispanic, NW = Non-White, O = Other, W = White

*ADHD* attention-deficit/hyperactivity disorder; *BFRB* Body Focused Repetitive Behavior; *CCHS* Canadian Community Health Survey; *CG* Comparison group; *CMTD* chronic multiple tic disorder (terminology used by Gadow et al., 2009, includes 62 TS and 4 “CMTD”); *CTD* chronic tic disorder; *DISC-IV* Diagnostic Interview Schedule for Children, fourth edition; *CVT* chronic vocal tics; *DSM* Diagnostic and Statistical Manual of Mental Disorders (*III-R* third edition, revised; *IV* fourth edition; *IV-TR* fourth edition, text-revised); *ICD* International Classification of Diseases; *MOVES* motor tic, obsession and compulsion, and vocal tic evaluation survey; *nc* not clear in paper; *nd* no date (years) presented for time of data collection; *NHIS-TS* National Hospital Interview Schedule for TS; *NSCH* National Survey of Children’s Health; *NS-DATA* National Survey of the Diagnosis and Treatment of ADHD and Tourette syndrome; *NR* Not Reported; *NSR* National School Register; *OCD* obsessive–compulsive disorder; *OCS* obsessive compulsive symptoms; *PTD* persistent tic disorder, including TS, chronic tic disorders, and persistent tic disorders; *SCID I* The Structured Clinical Interview for DSM-IV for Axis I diagnoses, patient edition (-P = parent edition); *TD* tic disorder, including transient/provisional; *TODS-PR* The Tourette Disorder Scale–Parent-Rated Version; *TS* Tourette syndrome; *TSA* Tourette Syndrome Association (Italy); *TSAA* Tourette Syndrome Association of Australia; *TSAV* Tourette Syndrome Association of Victoria; *US* United States; *UK* United Kingdom; *w/o* without

**Table 2:** Findings for broad indicators encompassing multiple domains from studies included in *Systematic Literature Review on Public Health Impacts of Persistent Tic Disorders: Education and Employment*

Category	Lead author, year	Indicator (mode of assessment)	Outcome, sample type, result	Sample age group
School-related quality of life	Cutler et al., 2009	PedsQL school functioning (C)	Mean score, TS vs. normative sample = 51.8 vs. 78.8^†^	Child/Adolescent
	Erbilgin Gun & Kilincaslan, 2019	PedsQL school functioning (P and C)	Mean score, TD + ADHD (P) vs. CG with ADHD only (P) = 47.55 vs. 57.17^†^ Mean score, TD + ADHD (C) vs. CG with ADHD only (C) = 52.84 vs. 66.55^†^	Child/Adolescent/Young Adult
	Gutierrez-Colina et al., 2015	PedsQL school functioning (P)	Mean score, TS vs. normative sample = 65.15 vs. 78.64^†^	Child/Adolescent
	Hao et al., 2010	PedsQL school functioning (C)	Mean score, TS vs. school-based CG = 70.35 vs. 85.21^‡^	Child
	Hesapcioglu et al., 2014	School-related Quality of Life Scale for Children (QoLS) (P and C)	Mean score, TS/CTD (P) vs. clinic patients without TS/CTD (P) = 66.5 vs. 80.7^†^ Mean score, TS/CTD (C) vs. clinic patients without TS/CTD (C) = 64.0 vs. 74.9^†^	Child/Adolescent
	Liu et al., 2017	ISLQ score for school life (C)	Mean score, TS vs. community CG = 5.38 vs. 6.31^†^	Child
	O’Hare et al., 2016	PedsQL school functioning (P)	Mean score, TS vs. community CG = 40.41 vs. 74.07^†^	Child/Adolescent
	Poh et al., 2018	PedsQL school functioning (P)	Mean score, CTD + ADHD vs. community CG with ADHD only = 38.7 vs. 43.4^††^	Child
	Storch et al., 2007	PedsQL school functioning (P and C)	Mean score, PTD (P) vs. population-based CG(P) = 61.78 vs. 85.47^†^ Mean score, PTD (C) vs. population-based CG(C) = 63.05 vs. 78.63^†^	Child/Adolescent
School competence	Balottin et al., 2016	YSR school competence scale (C)	Mean score, TS vs. community CG = 2.6 vs. 2.7^††^	Adolescent/Young Adult
	Gorman et al., 2010	CBCL school competence scale (P)	Mean score, TS vs. community CG = 40.7 vs. 50.2^†^	Adolescent/Young Adult
	Keenan et al., 2024	CBCL school competence scale (P)	Mean score, TS vs. community CG = 41.6 vs. 37.1^††^	Child
	Termine et al., 2006	CBCL school competence scale (P)	Mean score, TS vs. school-based CG = 45.4 vs. 52.5^†^	Child/Adolescent
	Zhu et al., 2006	CBCL school competence scale (P)	Mean score, TS vs. school-based CG = 4.14 vs. 4.80^†^	Child/Adolescent

Age Categories: Child =  < 12 years, Adolescent = 12–17 years, Young Adults = for sample groups that are 18–25 years old

Reporter types: C = child (includes adolescents), P = parent, T = teacher

*ADHD* attention-deficit/hyperactivity disorder; *CBCL* Child Behavior Checklist; *CG* comparison group; *CTD* chronic tic disorder; *ISLQ* Inventory of Subjective Life Quality; *PedsQL* Pediatric Quality of Life Inventory; *PTD* persistent tic disorder, including TS, chronic tic disorders, and persistent tic disorders; *TD* tic disorder; *TS* Tourette syndrome; *YSR* Youth Self-Report questionnaire

Significance: † = significant, †† = not significant, ‡ = significance not reported (or not reported specifically for groups compared here)

**Table 3:** School problems findings from studies included in *Systematic Literature Review on Public Health Impacts of Persistent Tic Disorders: Education and Employment*

Lead author, year	Indicator (mode of assessment)	Outcome, sample type, result	Sample age group
Balottin et al., 2016	MMPI-A (C) (lower scores are better)	Mean score for school problems, TS vs. community CG = 47.5 vs. 52.6^††^	Adolescent/Young Adult
Claussen et al., 2018	NSCH (P)	% contacted about school problems > 2 times in the past 12 months, TS vs. nationally representative CG = 37.4% vs. 12.5%^†^	Child/Adolescent
Jalenques et al., 2017	VSP-P (P)	% that had academic or other problems in school, TS vs. community CG = 79.7% vs. 24.0%^††^	Adolescent
Lin et al., 2012	SAICA (P and C) (lower scores are better for all categories)	Mean score for parent-report of “problems in school” (P), ADHD + TD vs. school-based CG = 1.67 vs. 1.14^†^ Mean score for self-report of “problems in school” (C), ADHD + TD vs. school-based CG = 1.69 vs. 1.30^‡^	Child/Adolescent

Age Categories: Child =  < 12 years, Adolescent = 12–17 years, Young Adults = for sample groups that are 18–25 years old

Reporter types: C = child (includes adolescents), P = parent

*ADHD* attention-deficit/hyperactivity disorder; *CG* comparison group; *MMPI-A* Minnesota Multiphasic Personality Inventory for Adolescents; *NSCH* National Survey of Children’s Health; *SAICA* Social Adjustment Inventory for Children and Adolescents; *TD* tic disorder; *TS* Tourette syndrome; *VSP-A/P* Vécu et Santé Perçue de l’Adolescent questionnaire (-P = parent version, -A = adolescent version)

Significance: † = significant, †† = not significant, ‡ = significance not reported (or not reported specifically for groups compared here)

**Table 4:** School impairment and limitations findings from studies included in *Systematic Literature Review on Public Health Impacts of Persistent Tic Disorders: Education and Employment*

Lead author, year	Indicator (mode of assessment)	Outcome, sample type, result	Sample age group
Cloes et al., 2017	Child Tourette Syndrome Impairment Scale (P and C)	Mean score for tic related impairment in school (P), TS vs. community/hospital CG = 0.62 vs. 0.23^†^ Mean score for tic related impairment in school (C), TS vs. community/hospital CG = 0.64 vs. 0.24^†^ Mean score for non-tic related impairment in school (P), TS vs. community/hospital CG = 0.91 vs. 0.23^†^ Mean score for non-tic related impairment in school (C), TS vs. community/hospital CG = 0.54 vs. 0.21^†^	Child/Adolescent
Ezpeleta & Toro, 2009	CAFAS (P and C)	% with high-moderate impairment or distress in school, TD without anxiety vs. ADHD without anxiety = 81.0% vs. 85.0%^‡^	Child/Adolescent
Pringsheim et al., 2009	Child Health Questionnaire (P)	Median for role/social limitations (Limitations in school, work, or activities with friends) due to problems with health, TS only group vs. National normative data = 100 vs. 100^††^ Median for role/social limitations (Limitations in school, work, or activities with friends) due to problems with emotion or behavior, TS only group vs. National normative data = 100 vs. 100^††^	Child/Adolescent

Age Categories: Child =  < 12 years, Adolescent = 12–17 years

Reporter types: C = child (includes adolescents), P = parent

*ADHD* attention-deficit/hyperactivity disorder; *CAFAS* Child and Adolescent Functioning Assessment Scale; *CG* comparison group; *TD* tic disorder; *TS* Tourette syndrome

Significance: † = significant, †† = not significant, ‡ = significance not reported (or not reported specifically for groups compared here)

**Table 5:** Academic performance and learning difficulties findings from studies included in *Systematic Literature Review on Public Health Impacts of Persistent Tic Disorders: Education and Employment*

Category	Lead author, year	Indicator (mode of assessment)	Outcome, sample type, result	Sample age group
Academic performance	Berg et al., 2024b	SARS CoV2 pandemic experiences survey	Median school performance during the COVID-19 pandemic, TS vs. neurotypical = 3.0 vs. 3.0^‡^ (out of 5, with higher number indicating poorer school performance)	Child/Adolescent/Young adult
	Claussen et al., 2018	NSCH (P)	% that do not complete all homework, TS vs. nationally representative CG = 27.3% vs. 13.3%^†^	Child/Adolescent
	Cubo et al., 2013	School data (T)	% with poor school performance, TD vs. school-based CG = 14.2% vs. 23.1%^†^ OR = 0.55, CI 0.32–0.93. p = 0.03 unadjusted OR (kids w/TD have lower odds of poor academic performance vs CG)^†^	Child/Adolescent
	Cubo et al., 2017	School data (trained rater)	Mean marks last trimester, TD vs. school CG = 5.7 vs. 6.3^†^	Child/Adolescent
	Horesh et al., 2018	BALES (C)	Mean positive school [academic] events in past year, TS vs. community CG = 6.54 vs. 5.79^‡^ Mean negative school [academic] events in past year, TS vs. community CG = 3.32 vs. 3.54^‡^	Child/Adolescent
	Jalenques et al., 2017	VSP-A (C)	Mean HRQoL dimension scores for school performance, TS vs. community CG = 56.6 vs. 62.8^††^	Adolescent
	Lanzi et al., 2004	School data (T)	% with normal school performance, TD vs. school CG = 59% vs. 85%^†^ % with mildly impaired school performance, TD vs. school CG = 23% vs. 11%^†^ % with severely impaired school performance, TD vs. school CG = 18% vs. 4%^†^	Child
	Poh et al., 2018	Wide Range Achievement Test-4 [WRAT-4] standardized scoring)	Z-score math computation, CTD + ADHD vs. ADHD only community CG = 86.9 vs. 87.4^††^ Z-score word reading, CTD + ADHD vs. ADHD only community CG = 95.8 vs. 94.4^††^	Child
	Ricketts et al., 2022a	NS-DATA (P)	% with problematic or somewhat problematic overall school performance, TS vs ADHD only = 3.7% vs. 45.7%^†^ % with problematic writing performance, TS vs ADHD only = 16.9% vs. 43.7%^†^ % with problematic mathematics performance, TS vs. ADHD only = 12.2% vs. 49.9%^†^ % with problematic handwriting, TS vs. ADHD = 45% vs. 40.7%^††^ % with problematic reading performance, TS + ADHD vs. ADHD Only = 40.3% vs. 39.8%^††^ *Note: Estimate was suppressed for problematic reading performance for the TS only group due to small cell sizes*	Child/Adolescent
	Wei, 2011	The School and Family Adjustment Questionnaire (P)	% with outstanding/above average academic performance, TS vs. school-based CG = 77.4% vs. 60.2%^‡^	Child
Pass rate and grade retention	Claussen et al., 2018	NSCH (P)	% had repeated a grade, TS vs. nationally representative CG = 20.2% vs. 9.8%^††^	Child/Adolescent
	Cubo et al., 2013	School data (T)	% pass rate over the last 3 months of elementary school, TD vs. school-based CG = 80.4% vs. 71.7%^††^ % pass rate over the last 3 months of middle and high school, TD vs. school-based CG = 17.3% vs. 18.9%^††^	Child/Adolescent
	Cubo et al., 2017	School data (trained rater)	% that experienced grade retention, TD vs. School-based CG = 16.4% vs. 8.3%^††^	Child/Adolescent
	Jalenques et al., 2017^34^	VSP-P (P)	% that had already repeated a year, TS vs. community CG = 37.3% vs. 10.7%^‡^	Adolescent
	Lund et al., 2023	Structured interview (C)	% that passed high school, TS vs. School-based CG = 64.6% vs. 74.5%^††^ % that passed lower-secondary school, TS vs. School-based CG = 80.5% vs. 97.9%^†^	Adolescent/Young Adult (longitudinal)
	Perez-Vigil et al., 2018	National School Register (school data)	% who passed core course: Swedish, PTD vs. non-PTD register population = 87.3% vs. 96.2%^†^ % who passed core course: English, PTD vs. non-PTD register population = 86.4% vs. 95.2%^†^ n (%) for those who passed core course: Mathematics, PTD vs. non-PTD register population = 84.0% vs. 94.2%^†^ *Note: All significance tests were based on odds ratios adjusted for sex, year of birth, maternal age, paternal age, and parity. Those with a PTD were also significantly less likely than those without a PTD to pass each of the 13 additional (non-core) courses, including arts, biology, chemistry, geography, handcraft textile/wood, history, home and consumer studies, knowledge of society, music, physics, religion, sports and health, and technology (PTD range: 70.7%−87.3%; non-PTD range: 90.6%–96.6%)*	Child/Adolescent/Adult (longitudinal)
Learning problems/difficulties	Cubo et al., 2013	School data (T)	% with learning difficulties, TD vs. school-based CG = 32.7% vs. 33.5%^††^	Child/Adolescent
	Jiang et al., 2025	Conners Parent Symptom Questionnaire (P) Weiss Functional Impairment Rating Scales (WFIRS) (P)	Learning problems Z-scores, Severe TD + ADHD vs. Severe ADHD, −0.93 vs. 0.73^‡^ Learning/school problems Z-scores, Severe TD + ADHD vs. Severe ADHD, −0.77 vs. 0.63^‡^	Child/Adolescent
	Khalifa & Knorring, 2005	Structured interview (P)	% with parent-report of “learning difficulties”, TS vs. transient tic community-based CG = 8% vs. 4%^‡^	Child/Adolescent
	Termine et al., 2022	Study-specific questionnaire (P)	t = −2.58 increased difficulties with remote learning during COVID lockdown, TD vs. children/adolescents without TD^†^	Child/Adolescent
	Watson et al., 2024	Conners-3 Parent Short Form (P)	Learning problems T-scores, TS vs. CG, 57 vs. 49^†^	Adolescent

Age Categories: Child =  < 12 years, Adolescent = 12–17 years, Young Adults = for sample groups that are 18–25 years old, Adult = 18 + years

Reporter types: C = child (includes adolescents), P = parent, T = teacher

*ADHD* attention-deficit/hyperactivity disorder; *BALES* Brief Adolescent Life Events Scale; *CG* comparison group; *CTD* Chronic Tic Disorder; *HRQoL* Health-Related Quality of Life; *NSCH* National Survey of Children’s Health; *NS-DATA* National Survey of the Diagnosis and Treatment of ADHD and Tourette syndrome; *PTD* persistent tic disorder, including TS, chronic tic disorders, and persistent tic disorders; *TD* tic disorder; *TS* Tourette syndrome; *VSP-A/P* Vécu et Santé Perçue de l’Adolescent questionnaire (-P = parent version, -A = adolescent version); *YSR* Youth Self-Report questionnaire

Significance: † = significant, †† = not significant, ‡ = significance not reported (or not reported specifically for groups compared here)

**Table 6:** Special education services and support findings from studies included in *Systematic Literature Review on Public Health Impacts of Persistent Tic Disorders: Education and Employment*

Lead author, year	Indicator (mode of assessment)	Outcome, sample type, result	Sample age group
Claussen et al., 2018	NSCH (P)	% that had an IEP, TS vs. nationally representative CG = 52.8% vs. 11.1%^†^	Child/Adolescent
Cubo et al., 2013	School data (T)	% with academic support at home, TD vs. school-based CG = 21.4% vs. 16.7%^††^ % with academic support at school, TD vs. school-based CG = 11.3% vs. 16.7%^††^	Child/Adolescent
Cubo et al., 2017	School data (trained rater)	% with academic support at school, TS vs. school-based CG = 11.4% vs. 10.3%^††^ % with psychological support at school, TS vs. school-based CG = 90.2% vs. 87.2%^††^	Child/Adolescent
Gadow et al., 2009	The Parent Questionnaire (P)	% currently in special education, CMTD + ADHD vs. community CG with ADHD only = 55% vs. 50%^††^ % received early childhood special education, CMTD + ADHD vs. community CG with ADHD only = 21% vs. 17%^††^	Child
Khalifa & Knorring, 2006	Structured interview (P)	% in full time special education, TS vs. school-based controls = 20% vs. 4%^††^ % in part time special education, TS vs. school-based controls = 48% vs. 8%^†^	Child/Adolescent

Age Categories: Child =  < 12 years, Adolescent = 12–17 years

*ADHD* attention-deficit/hyperactivity disorder; *CG* comparison group; *CMTD* chronic multiple tic disorder (terminology used by Gadow et al., 2009, includes 62 TS and 4 “CMTD”); *IEP* Individualized Education Plan; *TD* Tic disorder; *TS* Tourette syndrome

Significance: † = significant, †† = not significant, ‡ = significance not reported (or not reported specifically for groups compared here)

**Table 7:** School type and student status findings from studies included in *Systematic Literature Review on Public Health Impacts of Persistent Tic Disorders: Education and Employment*

Lead author, year	Indicator (mode of assessment)	Outcome, sample type, result	Sample age group
Chalita et al., 2012	School data (school administrative authorities)	Unadjusted OR for high school dropout among students with symptoms suggestive of a tic disorder vs. CG without symptoms suggestive of a psychiatric disorder = 4.23^†^	Adolescent
Claussen et al., 2018	NSCH (P)	% attending public school, TS vs. nationally representative CG = 80.4% vs. 87.8%^††^ % attending private school, TS vs. nationally representative CG = 8.2% vs. 10.0%^††^ % attending home school, TS vs. nationally representative CG = 11.4% vs. 2.3%^††^ % attending private or home school, TS vs. nationally representative CG = 19.6% vs. 12.3%^††^	Child/Adolescent
Cubo et al., 2013	School data (T)	% attending public schools, TS vs. school-based CG = 37.7% vs. 57.0%^†^ % attending catholic schools, TS vs. school-based CG = 62.3% vs. 43.4%^†^	Child/Adolescent
Cubo et al., 2017	School data (trained rater)	% attending public schools, TS vs. school-based CG = 57.4% vs. 47.4%^††^ % attending state-assisted [private] schools, TS vs. school-based CG = 42.6% vs. 53.6%^††^	Child/Adolescent
Debes et al., 2010	Study-specific interview questions (P & C together)	% changed school because of TS-related problems, TS vs. school-based CG = 23% vs. 6.9%^†^	Child/Adolescent/Adult
Zinna et al., 2021	Hospital data (hospital staff)	% that were not in education on admission to inpatient mental health unit, PTD vs. clinic-based CG = 48.8% vs. 39.4%^††^ % that were not in education on discharge from inpatient mental health unit, PTD vs. clinic-based CG = 4.7% vs. 5.1%^††^	Child

Age Categories: Child =  < 12 years, Adolescent = 12–17 years, Adult = 18 + years

Reporter types for mode of assessment: C = child (includes adolescents), P = parent, T = teacher

*CG* comparison group; *NSCH* National Survey of Children’s Health; *PTD* persistent tic disorder, including TS, chronic tic disorders, and persistent tic disorders; *TD* tic disorder; *TS* Tourette syndrome

Significance: † = significant, †† = not significant, ‡ = significance not reported (or not reported specifically for groups compared here)

**Table 8:** School absences findings from studies included in *Systematic Literature Review on Public Health Impacts of Persistent Tic Disorders: Education and Employment*

Lead author, year	Indicator (mode of assessment)	Outcome, sample type, result	Sample age group
Berg et al., 2024a	Self-report	% missed **school** or work due to tics, TS vs. FTLB = 31.8% vs. 62.9%^†^ Average % time absent school or work due to tics, TS vs. FTLB = 7.00 vs. 20.28^†^	Child/Adolescent/Young adult
Claussen et al., 2018	NSCH (P)	% that missed > 2 school days in the past 12 months, TS vs. nationally representative CG = 59.0% vs. 48.3%^††^	Child/Adolescent
Cubo et al., 2013	School data (T)	Mean number of school absences (in days), TS vs. school-based CG = 0.9 vs. 1.3^††^	Child/Adolescent
Guttmann-Steinmetz et al., 2009	CSI-4 conduct disorder symptom severity scale (T)	Mean score for “plays hooky”, CMTD + ADHD vs. community CG = 0.0 vs. 0.0^††^	Child

Age Categories: Child =  < 12 years, Adolescent = 12–17 years; Young adult = 18–25 years

Reporter types for mode of assessment: P = parent, T = teacher

*ADHD* attention-deficit/hyperactivity disorder; *CG* comparison group; *CMTD* chronic multiple tic disorder (terminology used by Gadow et al., 2009, includes 62 TS and 4 “CMTD”); *CSI-4* Child Symptom Inventory-4; *FTLB* Functional tic-like behavior; *NSCH* National Survey of Children’s Health; *TS* Tourette syndrome

Significance: † = significant, †† = not significant, ‡ = significance not reported (or not reported specifically for groups compared here)

**Table 9:** Attitudes about school findings from studies included in *Systematic Literature Review on Public Health Impacts of Persistent Tic Disorders: Education and Employment*

Lead author, year	Indicator (mode of assessment)	Outcome, sample type, result	Sample age group
Claussen et al., 2018	NSCH (P)	% that “do not care about doing well in school”, TS vs. nationally representative CG = 27.0% vs. 13.4%^††^	Child/Adolescent
Guttmann-Steinmetz et al., 2010	CSI-4 (P)	Mean score for “avoids school”, CMTD + ADHD vs. community CG = 0.33 vs. 0.14^††^	Child
Lin et al., 2012	SAICA (P and C) (lower scores = better for all categories)	Mean score for negative attitude toward school (P), ADHD + TD vs. school-based CG: 2.05 vs. 1.29^†^ Mean score for negative attitude toward school (C), ADHD + TD vs. school-based CG: 2.00 vs. 1.67^‡^	Child/Adolescent

Age Categories: Child =  < 12 years, Adolescent = 12–17 years

Reporter types for mode of assessment: C = child (includes adolescents), P = parent

*ADHD* attention-deficit/hyperactivity disorder; *CG* comparison group; Child/Adol = children and adolescents; *CMTD* chronic multiple tic disorder (terminology used by Gadow et al., 2009, includes 62 TS and 4 “CMTD”); *CSI-4* Child Symptom Inventory-4; *NSCH* National Survey of Children’s Health; *SAICA* Social Adjustment Inventory for Children and Adolescents; *TD* tic disorder; *TS* Tourette syndrome

Significance: † = significant, †† = not significant, ‡ = significance not reported (or not reported specifically for groups compared here)

**Table 10:** Levels and years of education findings from studies included in *Systematic Literature Review on Public Health Impacts of Persistent Tic Disorders: Education and Employment*

Category	Lead author, year	Mode of assessment	Outcome, sample type, result	Sample age group
Levels and years of education				
Education level	Drury et al., 2016	Self-report	% with no formal qualification, TS vs. CG = 5.0% vs. 0.0%^††^ % with GCSE or equivalent, TS vs. CG = 20.0% vs. 5.0%^††^ % with A levels or equivalent, TS vs. CG = 30.0% vs. 50.0%^††^ % with degree level or equivalent, TS vs. CG = 45.0% vs. 45.0%^††^	Adult
	Gomes de Alvarenga et al., 2012	Not clear	% with education level: illiterate, OCD + TD vs OCD–TD = 5.1% vs. 7.5%^††^ % with education level: elementary, OCD + TD vs. OCD–TD = 15.3% vs. 13.7%^††^ % with education level: high school, OCD + TD vs. OCD–TD = 49.6% vs. 47.2%^††^ % with education level: college undergraduate, OCD + TD vs. OCD–TD = 23.3% vs. 26.7%^††^ % with education level: college graduate, OCD + TD vs. OCD–TD = 6.8% vs. 4.9%^††^	Adult
	Muller-Vahl et al., 2020	Self-report	% with no school degree, CTD vs. CG = 2.4% vs. 0%^†^ % with certificate of secondary education, CTD vs. CG = 12.6% vs. 1.4%^†^ % with GCSE, CTD vs. CG = 28.3% vs. 17.4%^†^ % with general qualification of university entrance, CTD vs. CG = 29.1% vs. 42.6%^†^ % with university degree, CTD vs. CG = 27.6% vs. 38.6%^†^	Adult
	O’Connor et al., 2014	Self-report	% with secondary education or below, PTD vs. CG = 60.0% vs. 50.0%^††^ % with university education, PTD vs. CG = 40.0% vs 50.0%^††^	Adult
	Perez-Vigil et al., 2018	National School Register (school data) Swedish national population-based registers	Compulsory education: % eligible for vocational program, PTD vs. no PTD = 74.9% vs. 91.3%^†^ % eligible for academic program, PTD vs. no PTD = 56.3% vs. 77.0%^†^ Post compulsory education: % finishing upper secondary school, PTD vs. no PTD = 32.9% vs. 66.4%^†^ % starting a university degree, PTD vs. no PTD = 9.4% vs. 28.8%^†^ % finishing a university degree, PTD vs. no PTD = 3.1% vs. 13.9%^†^	Adult
	Ricketts et al., 2022b	Self-report	% with college or higher education, TD vs. CG = 100.0% vs. 100.0%^††^	Adult
	Worbe et al., 2011	Self-report	Mean education level, TS vs. CG = 2.3 vs. 3.2^††^ (Level 2 represents 14 years of study; level 3 represents 16 years of study)	Adult
	Yang et al., 2016	Self-report	% with no postsecondary degree, certificate, or diploma, TS vs general population = 68.3% vs. 36.3%^†^ (percentages reported for ages 12 years and older; significant difference for ages 18 years and older)	Adolescent/Adult
Years of education	Channon et al., 2003	Self-report	Mean years of education, TS vs. CG = 13.00 vs. 13.57^††^	Adult
	Channon et al., 2006	Self-report	Mean years of education, TS vs. CG = 13.25 vs. 13.04^‡^	Adult
	Channon et al., 2009	Self-report	Mean years of education, TS vs. CG = 14.19 vs. 13.78^††^	Adult
	Deckersbach et al., 2006	Self-report	Mean years of education, TS vs. CG = 14.9 vs. 14.5^††^	Adult
	Drury et al., 2016	Self-report	Mean years of education, TS vs. CG = 14.30 vs. 14.60^††^	Adult
	Eddy et al., 2010a	Self-report	Mean years of education, TS vs. CG = 12.83 vs. 13.40^‡^	Adolescent/Adult
	Eddy et al., 2010b	Self-report	Mean years of education, TS vs. CG = 12.94 vs. 14.63^‡^	Adult
	Eddy et al., 2011	Self-report	Mean years of education, TS vs. CG = 13.33 vs. 13.80^‡^	Adolescent/Adult
	Eddy et al., 2012	Self-report	Mean years of education, TS vs. CG = 13.55 vs. 14.35^‡^	Adolescent/Adult
	Eddy et al., 2014	Self-report	Mean years of education, TS vs. CG = 13.11 vs. 13.61^††^	Adolescent/Adult
	Eddy & Cavanna, 2015	Self-report	Mean years of education, TS vs. CG = 14.01 vs. 14.55^‡^	Adult
	Fan et al., 2018	Self-report	Mean years of education, TS vs. CG = 8.3 vs. 8.2^††^	Adult
	Kurvits et al., 2024	NR	Mean years of education, TD vs. CG = 15.9 vs. 17.8^††^	Adult
	Lavoie et al., 2007	Self-report	Mean years of education, TS/CTD vs. CG = 16.0 vs. 1.5^††^	Adult
	Moretto et al., 2011	Self-report	Mean years of education, TS vs. CG = 15.0 vs. 18.0^‡^	Adult
	Muller et al., 2003	Self-report	Mean years of education, TS + OCD = 12.4 (data not presented for controls, but reported that they did not differ from TS + OCD group)^††^	Adult
	Neuner et al., 2010	Self-report	Mean years of education, TS vs. CG = 11.9 vs. 13.8^‡^	Adult
	Palminteri et al., 2009	Self-report	Mean years of education, TS vs. CG = 11.3 vs. 15.1^‡^	Adult
	Rae et al., 2018	Self-report	Mean years of education, TS vs. CG = 15.0 vs. 14.0^††^	Adult
	Salvador et al., 2017	Self-report	Mean years of education, TS (non-medicated) vs. CG = 13.24 vs. 14.40^‡^	Adult
	Warren et al., 2020	Self-report	Mean years of education, TS vs. CG = 14.54 vs. 14.26^††^	Adult

Age Categories: Adolescent = 12–17 years, Adult = 18 + years

*A-Levels* (Advanced Level qualifications) are a subject-based qualification for students aged 16 and above in the United Kingdom (U.K.). They are usually studied over two years, leading to qualifications recognized for entrance to higher education institutes in the UK and many others worldwide. Most higher education institutes require a minimum of 3 subjects; *CG* comparison group; *CTD* chronic tic disorder; *GCSE* General certificate of secondary education; *NR* not reported; *OCD* obsessive–compulsive disorder; *PTD* persistent tic disorder, including TS, chronic tic disorders, and persistent tic disorders; *SAICA* Social Adjustment Inventory for Children and Adolescents; *TD* tic disorder; *TS* Tourette syndrome

Significance: † = significant, †† = not significant, ‡ = significance not reported (or not reported specifically for groups compared here)

**Table 11:** Employment findings from studies included in *Systematic Literature Review on Public Health Impacts of Persistent Tic Disorders: Education and Employment*

Lead author, year	Indicator (mode of assessment)	Outcome, sample type, result	Sample age group
Berg et al., 2024a	Self-report	% missed school or work due to tics, TS vs. FTLB = 31.8% vs. 62.9%^†^ Average % time absent school or work due to tics, TS vs. FTLB = 7.00 vs. 20.28^†^	Child/Adolescent/Young adult
Berg et al., 2024b	Self-report	Job loss during the COVID-19 pandemic, TS vs. neurotypical = 4.5% vs. 24.0%^‡^	Child/Adolescent/Yong adult
Colautti et al., 2023	Self-report	Profession, TS vs. healthy controls = 20.0% vs. 20.0% entrepreneurs and managers; 16.0% vs. 8.0% intellectual jobs; 20.0% vs. 36.0% generic jobs; 8.0% vs. 12.0% unemployed^††^ (note: 36.0% missing for TS, 24.0% missing for controls)	Adult
Ricketts et al., 2022b	Interview (S)	% employed, TD vs. community CG = 71.4% vs. 45.0%^††^ % students, TD vs. community CG = 21.4% vs. 50.0%^††^	Adult
Yang et al., 2016	Canada population survey of neuro-conditions (S)	% currently employed, TS vs. CG without TS = 46.4% vs. 67.7%^†^ % working full time, TS vs. CG without TS = 44.4% vs. 82.5%^†^ % working part time, TS vs. CG without TS = 55.4% vs. 17.5%^†^ Mean hours worked per week, TS vs. CG without TS = 29.1 vs. 39.6^†^ After restricting to ages 18 years and up and adjusting for age and sex, those with TS had significantly lower odds of: 1. Current employment (OR = 0.21, CI: 0.10–0.44)^†^ 2. Full time employment among those who were employed (OR = 0.16; CI: 0.05–0.52)^†^	Adolescent/Adult

Reporter types for mode of assessment: S = self

Age Categories: Adolescent = 12–17 years, Adult = 18 + years; Young adult = 18–25 years

*CG* comparison group; *FTLB* Functional tic-like behavior; *OR* odds ratio; *TD* tic disorder; *TS* Tourette syndrome

Significance: † = significant, †† = not significant, ‡ = significance not reported (or not reported specifically for groups compared here)

The following conventions were used during extraction. In articles with multiple potential comparison groups, we selected the group most like the general population. In articles with multiple PTD groups (e.g., TS only, TS and ADHD), we compared the TS only group to the non-PTD comparison group. For level and years of education outcomes, we only included primarily adult samples (ages ≥ 18 years) and excluded studies that matched on these outcomes. To facilitate comparison across studies, we extracted means and percentages; if neither was available, we coded the least adjusted results. We excluded six articles with overlap of participants and eligible outcomes, resulting in 167 articles with any outcome categories of interest eligible for inclusion; 62 articles included information on education or employment.

On March 5, 2025, we updated the literature review using the identical search strategy used in 2023. We reviewed the title and abstract of 917 articles published between July 6, 2023 and March 5, 2025 following the same process as the initial review. We excluded 584 articles during the title and abstract review and identified an additional 12 duplicates prior to full text review, resulting in 321 articles for full text review. In this stage, we excluded 250 articles, resulting in 71 articles for full text extraction, including seven that reported education and employment outcomes and had a comparison group without TS/PTD (Berg et al., 2024a, 2024b; Colautti et al., 2023; Jiang et al., 2025; Keenan et al., 2024; Kurvits et al., 2024; Watson et al., 2024). Two reviewers independently extracted data for study characteristics, education outcomes, and study quality for these seven papers; discrepancies were reviewed and discussed for final consensus. The findings for these studies are incorporated with the findings for the original search, for a total of 69 included papers.

## Results

The 69 included studies are summarized in Table 1. Nearly two-thirds (45) of the studies reported on TS only (versus including other tic disorders). To identify tic disorders, 51 studies used clinical assessment, 2 used ICD codes in healthcare data, three relied on parent-report and three relied on self-report of a previous diagnosis, and one study used teacher-report of symptoms related to diagnostic criteria. Three studies included individuals with a previous diagnosis with specific criteria (e.g., DSM-5) mentioned and the remaining 6 studies included individuals with a previous diagnosis, but did not provide additional detail. Most (55) studies included less than 100, and seven studies included more than 200, individuals with TS/PTD. Thirty-six studies primarily reported outcomes for children and adolescents (< 18 years), 26 studies primarily reported on adults (≥ 18 years) and 7 studies reported on adolescents (13–17 years) and adults. The TS/PTD group was predominantly male in most studies (over 60% male in 55 studies; in seven studies the sex distribution of the TS/PTD group was unclear or not reported). Most (51) studies did not report on race or ethnicity; of those that did, most samples (14) were > 80% White, one U.S. study sample was 10.4% minority (i.e., Black, Hispanic, or other) (Ricketts et al., 2022a), another U.S. study sample was 21.4% “minority” (not further defined) (Ricketts et al., 2022b), and one study based in Mexico described the sample as having “no significant racial-ethnic diversity” (Chalita et al., 2012). Individuals with TS/PTD were identified through: TS specialty clinics (22), other clinics (22), national surveys (3), schools (7), community-based settings (2), patient support organizations (1), a combination of clinic and patient support organizations (4), a combination of clinic- and community-based settings and patient support organizations (1), a specialty summer camp (1), and registry data (1); in 6 studies the recruitment population was not clearly described. Most studies took place in Europe (14 U.K., 5 Italy, 5 Germany, 4 France, 3 Sweden, 3 Spain, 2 Denmark, 1 Ireland, 1 Netherlands) or North America (12 U.S., 5 Canada, 1 U.S. and Canada, 1 Mexico). Others took place in China (4), Australia (2), Taiwan (2), Turkey (2), Brazil (1), and Israel (1).

The identified literature included a wide array of education outcomes, including parent and self-report of experiences at school and academic performance, validated scales (e.g., Child Behavior Checklist (CBCL), Pediatric Quality of Life Inventory [PedsQL]), and school data. These included specific, single-domain indicators and, composite indicators encompassing multiple domains (multi-domain indicators). Only two studies included employment outcomes. Outcomes are summarized in 10 categories based on labels, measures, and definitions used in the studies (Tables 2, 3, 4, 5, 6, 7, 8, 9, 10, 11): (1) multi-domain indicators (school-related quality of life, school competence), (2) school problems, (3) general school impairment and limitations, (4) academic performance and learning (academic performance, pass rate and grade retention, learning problems/difficulties), (5) special education services and supports, (6) school type and student status, (7) school absences, (8) attitudes about school, (9) level/years of education, and (10) employment status. To synthesize results by category we focused on statistically significant differences between TS/PTD and comparison groups; given the small sample sizes for many studies, we also noted differences in magnitude even when results were not significant or when statistical significance was not reported.

### Multi-Domain Indicators

#### School-Related Quality of Life (QoL)

Nine studies included parent and/or child report of school-related QoL, with composites inclusive of indicators such as school problems, levels of satisfaction, or school functioning (not presented separately in studies; Table 2) (Cutler et al., 2009; Erbilgin Gun & Kilincaslan, 2019; Gutierrez-Colina et al., 2015; Hao et al., 2010; Hesapcioglu et al., 2014; Liu et al., 2017; O'Hare et al., 2016; Poh et al., 2018; Storch et al., 2007). Seven studies used the PedsQL (Cutler et al., 2009; Erbilgin Gun & Kilincaslan, 2019; Gutierrez-Colina et al., 2015; Hao et al., 2010; O'Hare et al., 2016; Poh et al., 2018; Storch et al., 2007). All nine studies reported lower school-related QoL among children with TS/PTD compared to those without, regardless of the reporter (parent or self-report), study location, or QoL measure used. Group differences for QoL were statistically significant when individuals with TS/PTD were compared to national norms (Cutler et al., 2009; Gutierrez-Colina et al., 2015), population-based controls (Storch et al., 2007), or community controls (Erbilgin Gun & Kilincaslan, 2019; Hao et al., 2010; Hesapcioglu et al., 2014); group differences were not statistically significant in one of two studies comparing children with TS/PTD and ADHD to an ADHD-only comparison group (Poh et al., 2018).

#### School Competence

Four clinic-based studies and one study that recruited through multiple settings (e.g., clinic, community, patient organizations) reported on school competence (Table 2) based on the parent-rated CBCL or corresponding Youth Self Report (YSR) (Balottin et al., 2016; Gorman et al., 2010; Keenan et al., 2024; Termine et al., 2006; Zhu et al., 2006). Items contributing to school competence subscales include academic performance, special education, grade retention, and school problems (not presented separately) (Achenbach & Edelbrock, 1981). Three parent-report studies found lower mean scores for children or adolescents with TS compared to community controls (Table 2) (Gorman et al., 2010; Termine et al., 2006; Zhu et al., 2006), while the study that recruited through multiple settings found no difference in mean scores based on parent-report for children and adolescents with TS compared to community controls (Keenan et al., 2024) and one YSR study found no difference in school competence among adolescents with and without TS (Balottin et al., 2016).

### School Problems

Four studies reported on school problems (Table 3) (Balottin et al., 2016; Claussen et al., 2018; Jalenques et al., 2017; Lin et al., 2012). In three studies, parents of children or adolescents with TS reported more school problems compared to parents of children without TS; (Claussen et al., 2018; Jalenques et al., 2017; Lin et al., 2012) the difference was not statistically significant in one study (Jalenques et al., 2017). Results were less consistent for self-reported measures of school problems in two studies; children and/or adolescents with TS reported more school problems on the Social Adjustment Inventory for Children and Adolescents (although statistical testing was not reported), and similar school problems on the Minnesota Multiphasic Personality Inventory for Adolescents, compared to those without TS (Balottin et al., 2016; Lin et al., 2012).

### School Impairment and Limitations

Results were mixed across three studies examining general school impairment and limitations (Table 4) (Cloes et al., 2017; Ezpeleta & Toro, 2009; Pringsheim et al., 2009). One study reported no differences between children with TS/PTD and those without on limitations in schoolwork or activities due to health or behavior problems on the Child Health Questionnaire (Pringsheim et al., 2009). Another study reported that students with TS/PTD were somewhat less likely to experience high to moderate impairment or distress at school on the Child and Adolescent Functional Assessment Scale compared to students with ADHD, although statistical significance was not reported (Ezpeleta & Toro, 2009). Conversely, a study using the Child TS Impairment Scale found children with TS/PTD were more likely than children without TS/PTD to experience tic- and non-tic related impairment in school (Cloes et al., 2017).

### Academic Performance and Learning Difficulties

#### Academic Performance

Findings were mixed across and within ten studies reporting on academic or school performance (Table 5). Three studies reported that children and adolescents with TS/PTD had worse outcomes compared to those without TS/PTD (higher percentage with impaired performance; lower mean marks; higher percentage not completing homework) (Claussen et al., 2018; Cubo et al., 2017; Lanzi et al., 2004). However, three studies reported that children and adolescents with TS/PTD had better outcomes than those without TS/PTD. Two of these studies reported better academic performance among children with TS/PTD compared to school-based control groups (Cubo et al., 2013; Wei, 2011). The third reported lower overall problematic school performance, and lower problematic writing performance and mathematics performance among children with TS compared to children with ADHD (Ricketts et al., 2022a). Four studies reported similar outcomes to the comparison groups (problematic handwriting and reading school performance; performance on Wide Range Achievement Test-4; Vécu et Santé Perçue de l’Adolescent questionnaire school performance scores; Brief Adolescent Life Events Scale positive and negative school [academic] events) (Horesh et al., 2018; Jalenques et al., 2017; Poh et al., 2018; Ricketts et al., 2022a), although statistical testing was not reported for one of these outcomes (Horesh et al., 2018). Finally, one study reported similar school performance during the COVID-19 pandemic between children and adolescents with TS compared to neurotypical controls (Berg et al., 2024b).

#### Pass Rate and Grade Retention

Six studies reported on pass rates or grade retention (Table 5). While in five studies children with TS/PTD had lower pass rates or a higher percentage who had repeated a grade compared to those without TS/PTD (Claussen et al., 2018; Cubo et al., 2017; Jalenques et al., 2017; Lund et al., 2023; Perez-Vigil et al., 2018), results were statistically significant for only two of these studies (Lund et al., 2023; Perez-Vigil et al., 2018). One school-based study reported similar pass rates for children and adolescents with tic disorders for middle and high school, and a higher, but non-significant, pass rate for children with tic disorders for elementary school, compared to those without tic disorders (Cubo et al., 2013).

#### Learning Problems/Difficulties

Five studies reported on learning problems or learning difficulties (Table 5). One school-based study reported similar prevalence of learning difficulties among students with and without TS/PTD (Cubo et al., 2013). One clinic-based study reported significantly higher learning problems among adolescents with TS compared to controls (Watson et al., 2024) and a second study reported twice the prevalence of learning difficulties among those with TS compared to those with transient tics, however, in this second study prevalence was low in both groups and statistical significance was not reported (Khalifa & von Knorring, 2005). One study compared children with severe tic disorders and co-occurring ADHD to children with severe ADHD (and no tic disorder) and reported higher learning problems among children with severe ADHD compared to those with severe TD and ADHD using two different rating scales (Jiang et al., 2025). Finally, one study found that children and adolescents with tic disorders experienced more difficulties with remote learning during the COVID-19 pandemic lockdown compared to those without tic disorders (Termine et al., 2022).

### Special Education Services and Support

Five studies reported on special education or other school supports, including early intervention, having an individual education plan (IEP), academic support at home and school, and psychological support at school (Table 6). In two studies, a higher percentage of children and adolescents with TS had an IEP or were enrolled in part time special education compared to those without TS (Claussen et al., 2018; Khalifa & Von Knorring, 2006); one of these studies reported no difference for full time special education enrollment (Khalifa & Von Knorring, 2006). Three studies reported a similar percentage of children and/or adolescents with or without TS/PTD accessed special education or other school support; two of these studies were school-based and the other compared children with “chronic multiple tic disorder” and ADHD to children with ADHD only (Cubo et al., 2013, 2017; Gadow et al., 2009); one of these studies reported on special education as a demographic characteristic rather than a main outcome (Gadow et al., 2009).

### School Type and Student Status

Six studies reported on school type or student status (e.g., enrolled in school; Table 7). Results were mixed. In two studies, a smaller percentage of children with TS/PTD attended public school and a larger percentage of children with TS/PTD were enrolled in private school or homeschool compared to children without TS/PTD; for one of these studies, differences were not statistically significant (Claussen et al., 2018; Cubo et al., 2013). One study reported a higher percentage of students with TS/PTD attending public school, and a lower percentage attending state-assisted [private] schools, although results were not statistically significant (Cubo et al., 2017). In two studies, children and/or adolescents with TS/PTD were more likely to have changed schools (Debes et al., 2010) or dropped out of school (Chalita et al., 2012) compared to those without TS/PTD. In another study, a greater percentage of children with TS/PTD admitted to an inpatient pediatric mental health unit were not attending school prior to admission compared to those without TS/PTD, although this difference was not statistically significant (Zinna et al., 2021).

### School Absences

Three studies reported no statistically significant differences in school absences among children with and without TS/PTD (Table 8) (Claussen et al., 2018; Cubo et al., 2013; Guttmann-Steinmetz et al., 2009). Indicators included teacher report of “plays hooky”, mean number of school absences (Cubo et al., 2013; Guttmann-Steinmetz et al., 2009), and parent-report of greater than 2 school days missed in the past 12 months (Claussen et al., 2018). One study compared self-report of missed school/work among a mixed-age (child, adolescent, adult) sample of individuals with TS and functional tic-like behavior (FTLB) and found that a smaller percentage of individuals with TS missed school orwork compared to those with FTLB; similarly, individuals with TS missed fewer days of school orwork, on average, compared to those with FTLB (Berg et al., 2024a).

### Attitudes About School

Three studies reported on indicators of attitudes about school (Table 9). Indicators included whether child avoided school (Guttmann-Steinmetz et al., 2010), had a negative attitude toward school (Lin et al., 2012), and cared about doing well at school (Claussen et al., 2018); all included parent-report and one also included child report. Although results generally indicated that negative attitudes were more prevalent among students with TS/PTD (Claussen et al., 2018; Guttmann-Steinmetz et al., 2010; Lin et al., 2012), this difference was only statistically significant in one study, and for parent-report only (statistical significance was not reported for child report) (Lin et al., 2012).

### Education Level Completed and Years of Education

Eight studies reported on education level and 21 studies reported on years of education in primarily adult samples (Table 10). Most studies reported these indicators as part of the sample description; two studies reported on education level as a main finding (Perez-Vigil et al., 2018; Yang et al., 2016). In many studies the comparison group was recruited from an academic setting (see Table 1), which may bias the results.

The findings were mixed across nine studies that reported on education level. Six studies reported lower levels of education for adults with compared to without TS/PTD (Drury et al., 2016; Muller-Vahl et al., 2020; O'Connor et al., 2014; Perez-Vigil et al., 2018; Worbe et al., 2011; Yang et al., 2016), although differences were not significant in three studies with small samples (range = 14–60 with TS/PTD) (Drury et al., 2016; O'Connor et al., 2014; Worbe et al., 2011). Two studies reported similar education level among adults with and without TS/PTD, however one of these had a small sample of adults with TS/PTD (*n* = 14) (Ricketts et al., 2022b), and the other compared those with OCD + TD to OCD-TD (Gomes de Alvarenga et al., 2012). Both studies that included education level as a main finding reported lower education level among individuals with TS/PTD compared to without TS/PTD (Perez-Vigil et al., 2018; Yang et al., 2016).

Most (*n* = 13) of the 21 studies that reported years of education showed similar results for adults with and without TS/PTD (Channon et al., 2003, 2006, 2009; Deckersbach et al., 2006; Drury et al., 2016; Eddy & Cavanna, 2015; Eddy et al., 2010a, 2011, 2012, 2014; Fan et al., 2018; Muller et al., 2003; Warren et al., 2020). Six studies documented that adults with TS/PTD had at least one less year of education compared to adults without TS/PTD, but either the findings were not statistically significant, or statistical comparisons were not reported (Eddy et al., 2010b; Kurvits et al., 2024; Moretto et al., 2011; Neuner et al., 2010; Palminteri et al., 2009; Salvador et al., 2017). Two studies reported that adults with TS/PTD had approximately 1 year more of education than adults without TS/PTD, but the difference was not significant (Lavoie et al., 2007; Rae et al., 2018). Sample sizes of adults with TS/PTD were small across all 21 studies (range: 12–40).

### Employment Status

Five studies reported mixed results on employment status, including missed work, among adults with TS/PTD (Table 11). In a nationally representative Canadian sample, adults with TS had lower odds of any current employment and current full time employment compared to the general population (Yang et al., 2016). Four studies showed that individuals with tic disorders were more likely to be employed or less likely to be unemployed or to miss work than individuals in the comparison groups. A U.S. clinic-based study (*n* = 14 adults with TS) found more adults with TS were employed and fewer were enrolled as students than age- and sex-matched controls, although these differences were not statistically significant (Ricketts et al., 2022b). In an Italian study, compared to healthy controls, 30 adults with TS were less likely to be unemployed, more likely to have "intellectual" jobs, and less likely to have "generic" jobs, but the differences were not significant (Colautti et al., 2023). In a Canadian study describing job loss during the COVID-19 pandemic, 22 individuals (aged 11–25 years) with TS were less likely to report job loss compared to neurotypical controls, although a statistical comparison was not reported (Berg et al., 2024b). The fourth study compared employment outcomes between individuals with tic disorders to individuals with a functional tic-like behavior (FTLB) in Canada and reported that individuals aged 11–25 years with TS were significantly less likely to miss school or work compared to individuals with a FTLB (this identical finding was also reported in the section above on school absences, and Table 8) (Berg et al., 2024a). Although four studies showed better employment outcomes among individuals with tic disorders compared to the comparison group, each study included 30 or fewer individuals with tic disorders, the differences were not significant in two studies and a statistical comparison was not presented in the third, and the fourth study compared individuals with TS to individuals with FTLB and the outcome included both missed school and missed work (Berg et al., 2024a, 2024b; Colautti et al., 2023; Ricketts et al., 2022b). In contrast, the one study that showed that adults with tic disorders had lower employment included a nationally representative sample including 122 individuals with TS, and the finding was significant (Yang et al., 2016).

### Study Quality

We rated study quality using the Newcastle–Ottawa Scale for 23 case–control studies and 42 cohort studies (4 studies included a sample where 100% of individuals had another disorder and were not rated for quality; see Supplemental information). Case control studies generally had low risk of bias for having an adequate case definition based on independent validation, and cohort studies generally had low risk of bias for how representative the tic disorder group was for the average person with TS/PTD; both were part of the selection criteria. Case control studies generally scored as having a high risk for bias for non-response rate (with studies not reporting a response rate scoring 0) and cohort studies generally scored as having a high risk of bias for the adequacy of follow-up groups, based on retention of 80% of the sample (with cross-sectional studies scoring 0). The three studies with the lowest risk of bias were Gorman et al., 2010, Lund et al., 2023, and Perez-Vigil et al., 2018. Gorman et al., 2010 was one of three studies that reported lower mean scores of school competence using the CBCL for children or adolescents with TS compared to community controls (Gorman et al., 2010). Lund et al., 2023 and Perez-Vigil et al., 2018 were among five studies that reported significant associations between having TS/PTD and lower pass rates (or repeating a grade) (Lund et al., 2023; Perez-Vigil et al., 2018). In addition, Perez-Vigil was one of six studies that reported significantly lower levels of education for adults with compared to without TS/PTD (Perez-Vigil et al., 2018).

## Discussion

Based on evidence included in this review, children and adolescents with TS/PTD may experience poorer school-related QoL, lower school competence, and more parent-reported school problems compared to those without TS/PTD. Although findings were not always statistically significant, children and adolescents with TS/PTD may also have poorer attitudes about school and lower pass rates/more frequent grade retention; individual studies showed children and adolescents with TS/PTD were more likely than those without to attend private school, homeschool, change schools, drop out, or not be enrolled.

Results for education level completed, years of education, and employment status among adults with TS/PTD were equivocal. For level and years of education, although we excluded studies that described matching on education, other aspects of study design, including recruitment of controls, may have influenced the findings. Future research on this topic would benefit from larger sample sizes and additional data on employment among adults with TS/PTD.

Academic achievement and school engagement can be influenced by an array of intersecting multi-level factors that can result in educational and health disparities, including for students with disabilities (Kuhfeld et al., 2018; McKinley Yoder & Cantrell, 2019; Zajacova & Lawrence, 2018). Lower school-related QoL and school competence, more school problems, and more negative attitudes about school among students with TS/PTD may reflect the disproportionate role of social, environmental, and institutional factors in shaping their school experiences and opportunities. While students with TS/PTD may experience academic and learning challenges due to tics (e.g., fatigue due to suppressing tics, handwriting difficulties), many also face barriers at school in the form of discipline practices, lack of accommodations, and challenges related to stigma and bullying (Mingbunjerdsuk & Zinner, 2020). School-related social outcomes, which may interact with school and academic experiences, were coded within the social domain for our literature review and are not included in this paper (Wong et al., 2022).

Results were mixed for special education or other school supports; students with TS more often accessed special education or had an IEP compared to those without (Claussen et al., 2018; Khalifa & Von Knorring, 2006), whereas a similar percentage of students with any tic disorder accessed these services compared to those without a tic disorder (Cubo et al., 2013, 2017). In one study of children with ADHD, a similar percentage of those who also had a PTD accessed special education services compared to those with ADHD only (Gadow et al., 2009). Ensuring access to education for children with disabilities is recognized internationally^1^ by legal protections. In the United States, the Individuals with Disabilities Education Act legislates access to a free and appropriate public education for children with disabilities.^2^ Although not all students with TS/PTD will require school services, U.S. students with TS/PTD who experience limitations or impairment with learning may qualify for services such as special education, counseling, and occupational therapy via an individualized education program (IEP) and accommodations under the “Other Health Impairment” category due to having TS, or due to other co-occurring conditions such as learning disabilities, speech or language disorders (Mingbunjerdsuk & Zinner, 2020; Simpson et al., 2020). Children with TS/PTD, in the U.S., may also qualify for educational accommodations (e.g., extra time to complete classwork, organizational supports, supportive technology) through a Sect. 504 plan^3^ but national data for Sect. 504 plans by condition are not available. Our findings may point to differential access to special education services by specific TS/PTD diagnosis, variability in special education eligibility criteria, or educational accommodations for students with TS/PTD not captured by these studies. In addition, the studies included report on the prevalence of special education services and supports among all individuals with TS/PTD, rather than distinguishing the prevalence of these services among those who need them.

Schools have the potential to positively influence health trajectories across the life course (Wong et al., 2022), including for individuals with TS/PTD. School interventions and supports can help address the needs of students, provide access to protective and health promotive factors and safe and supportive learning environments, and foster social and school connectedness (Mingbunjerdsuk & Zinner, 2020). Similarly, employment is positively associated with health outcomes (Hergenrather et al., 2015). Healthcare providers can collaborate with patients, their families, and their schools to support positive school and work experiences among individuals with TS/PTD; they can engage with patients and their families about functional, social, or academic challenges at school and work, and share information on legal rights to accommodations or other supports to optimize functioning at school and work (Mingbunjerdsuk & Zinner, 2020; Pringsheim et al., 2019). Healthcare providers can also engage school personnel directly to share information about symptoms of TS/PTD and co-occurring disorders to increase awareness and understanding, and to recommend specific school accommodations to help mitigate the academic and functional impact of tics, co-occurring disorders, and stigma (Mingbunjerdsuk & Zinner, 2020). Additionally, healthcare providers can support interventions that promote positive behavior, such as by sharing resources with schools on the benefits of positive discipline for children with TS/PTD.^4^ The Tourette Association of America (TAA) offers tools to help healthcare providers, school personnel, and families identify accommodations that may address specific student and work-related needs.^5^ Trainings for healthcare providers are available through the TAA and the American Academy of Pediatrics to learn about TS/PTD and how to help patients and their families access school and work accommodations and supports.^6^

The conclusions of this review are limited to the types of studies included which used relatively small (most *n* < 100) and homogeneous (e.g., primarily White individuals with TS/PTD) samples. Although most studies took place in Europe (38) or North America (19), studies from Asia, Australia, and South America were included. No differences were observed by country or region; however, country-specific education systems, policies, and resources may impact these outcomes. Approximately two-thirds of studies only included participants with TS (rather than PTDs). A majority (50) of studies identified individuals with TS/PTD using clinical assessment. Quality ratings using the Newcastle–Ottawa Scale (NOS) tended to be low, suggesting potential bias across studies; however, there are limitations associated with the NOS. For example, cross-sectional studies all receive scores of 0 for time to follow-up, part of the outcome criteria for cohort studies (Luchini et al., 2017). In addition, most of the studies included in our review did not report a response rate, resulting in lower NOS scores. Finally, while many studies included in our review used a comprehensive assessment of tic disorders following established criteria, most received a score of 0 for ascertainment of tics because the assessment was not blinded. Blind rating is difficult for disorders with clear visual or auditory signs like TS/PTD. Other quality rating scales have similar or related limitations, including lack of reliability between reviewers (Armijo-Olivo et al., 2012; Luchini et al., 2017; Margulis et al., 2014; Moskalewicz & Oremus, 2020). To better document the association between TS/PTD and education and employment outcomes, future studies can include larger, more heterogeneous samples and include outcomes related to employment and transition to higher education. Longitudinal studies could assess trajectories in educational and employment outcomes and their association with other life outcomes in TS/PTD. Study quality could be improved by ensuring representativeness of cases (e.g., through recruitment of consecutive patients vs. convenience samples), documentation of response rates, and improved comparability through recruitment strategies and/or adjustment for confounding factors, such as the presence of co-occurring mental disorders.

There are additional limitations to consider in the interpretation of our findings. First, we excluded studies without comparison groups which may have limited the types and number of outcomes summarized. Second, our focus on unadjusted/least adjusted findings precluded us from examining the impact of co-occurring disorders or severity of TS/PTD on educational outcomes. Co-occurring disorders and severity of TS/PTD are known to influence this relationship, and co-occurring disorders may be the primary cause of educational challenges (Claussen et al., 2018; Cubo et al., 2013; Debes et al., 2010; Jiang et al., 2025; Keenan et al., 2024); however, our findings represent the general association between TS/PTD and educational outcomes. Finally, some education outcomes were reported as demographic or contextual factors rather than main outcomes.

## Conclusion

This systematic review describes the available evidence on education and employment outcomes among individuals with TS/PTD. Children and adolescents with TS/PTD may experience poorer school-related QoL, lower school competence, and more parent-reported school problems compared to those without TS/PTD. Future research with larger, more heterogeneous samples, and on the transition from K-12 to secondary education or employment for individuals with TS/PTD, may help address identified research gaps. Findings from this study can be used by healthcare providers and school personnel to inform supports for students with TS/PTD.

## Supplementary Information

Below is the link to the electronic supplementary material.

Supplementary file1 (PDF 512 KB)

## Footnotes

1 https://social.desa.un.org/issues/disability/crpd/article-24-education

2 https://sites.ed.gov/idea/statute-chapter-33, https://sites.ed.gov/idea/statuteregulations/

3 https://www.ecfr.gov/current/title-10/chapter-I/part-4/subpart-B/

4 https://www.changelabsolutions.org/sites/default/files/2024-07/CMH-PositiveSchoolDiscipline-Factsheet-FINAL_ACCESS-20240701.pdf

5 https://tourette.org/resources/back-to-school-resources/, https://tourette.org/wp-content/uploads/TAA_Young_Adult_Toolkit.pdf

6 https://tourette.org/taa-school-education-video-series/, https://www.aap.org/Tourette-Syndrome-Course-Series
