# Supplementary material for: Systematic Literature Review on Public Health Impacts of Persistent Tic Disorders: Education and Employment
Source: Clin Child Fam Psychol Rev. 2026 Jan 3;29(1):1–32. doi: 10.1007/s10567-025-00537-3 (PMC12979344; doi:10.1007/s10567-025-00537-3)
Supplement: Supplementary file 1 — Supplementary file1 (PDF 512 KB) [file 10567_2025_537_MOESM1_ESM.pdf]

Supplemental information for: Systematic Literature Review on Public Health Impacts of Persistent Tic Disorders: Education and Employment, Clinical Child and Family Psychology Review.

Helena J. Hutchins[1], Patricia Whalen[1,2], Jorge Verlenden[3], Hidayat Ogunsola,[1,2] Brooke S. Staley[1,4], Rebecca T. Leeb[1], Wendy Wegman[5], Rebecca H. Bitsko[1]

[1]Applied Research and Evaluation Team, Division of Human Development and Disability, National Center on Birth Defects and Developmental Disabilities, Centers for Disease Control and Prevention, 4770 Buford Hwy S106-4, Atlanta, GA 30341-3717, USA.

[2] Oak Ridge Institute for Science and Education, CDC Research Participation Programs, Oak Ridge, TN, USA

[3] Division of Adolescent and School Health, National Center for Chronic Disease Prevention and Health Promotion, Centers for Disease Control and Prevention, 4770 Buford Hwy, Atlanta, GA

[4] Epidemic Intelligence Service, CDC, Atlanta, GA, USA

[5] Tourette Association of America, 42-40 Bell Boulevard, Suite 507, Bayside, NY 11361

Corresponding author: Helena J. Hutchins, MPH, BSEd; pne8@cdc.gov

Newcastle-Ottawa Scale quality assessment of studies included in "Systematic Literature Review on Public Health Impacts of Persistent Tic Disorders: Education and Employment" -- Case-Control Studies

Reference	Selection	Comparability	Outcome
Berg, 2024a	Some	High	High
Berg, 2024b	Low	Some	High
Balottin, 2016	Low	Low	High
Colautti, 2023	Low	Some	High
Drury, 2016	Some	High	High
Gorman, 2010	Low	Low	Low
Jalenques, 2017	High	High	High
Khalifa, 2005	Some	High	High
Khalifa, 2006	Low	High	High
Kurvits, 2024	High	High	High
Lin, 2012	High	Low	High
Moretto, 2011	Some	High	High
Muller, 2003	Some	High	High
Neuner, 2010	Some	High	High
O'Hare, 2016	Some	High	High
Palminteri, 2009	High	High	High
Ricketts, 2022b	Low	High	High
Termine, 2006	Low	High	High
Termine, 2022	High	High	High
Warren, 2020	Low	High	High
Watson, 2024	Low	Some	High
Wei, 2011	Some	High	High
Zhu, 2006	Low	High	High

Risk of Bias
- Low
- Some
- High

Newcastle-Ottawa Scale quality assessment of studies included in "Systematic Literature Review on Public Health Impacts of Persistent Tic Disorders: Education and Employment" -- Cohort Studies

Reference	Selection	Comparability	Outcome
Chalita, 2012	High	Some	High
Channon, 2003	Some	High	High
Channon, 2006	Some	High	High
Channon, 2009	Some	High	High
Claussen, 2018	Some	Low	High
Cloes, 2017	Some	High	High
Cubo, 2013	Low	Some	High
Cubo, 2017	Low	High	High
Cutler, 2009	Some	High	High
Debes, 2010	Low	Some	High
Deckersbach, 2006	Low	High	High
Eddy, 2010a	Some	High	High
Eddy, 2010b	Some	High	High
Eddy, 2011	Some	High	High
Eddy, 2012	Some	High	High
Eddy, 2014	Low	High	High
Eddy, 2015	Some	High	High
Ezpeleta, 2009	Low	Some	High
Fan, 2018	Some	High	High
Gutierrez-Colina, 2015	High	High	High
Guttmann-Steinmetz, 2009	Low	High	High

Reference	Selection	Comparability	Outcome
Guttmann-Steinmetz, 2010	Low	High	High
Hao, 2010	Some	Some	High
Hesapcioglu, 2014	Some	Some	High
Horesh, 2018	Low	High	High
Jiang, 2025	Some	Some	High
Keenan, 2024	Some	Some	High
Lanzi, 2004	Low	High	High
Lavoie, 2007	Low	High	High
Liu, 2017	Low	High	High
Lund, 2023	High	High	High
Muller-Vahl, 2020	Low	High	High
O'Connor, 2014	Some	High	High
Perez-Vigil, 2018	Low	Some	Some
Pringsheim, 2009	High	High	High
Rae, 2018	Low	High	High
Ricketts, 2022a	Low	Some	Some
Salvador, 2017	Some	Some	High
Storch, 2007	Some	High	High
Worbe, 2011	Some	Some	High
Yang, 2016	Some	High	High
Zinna, 2021	Some	High	High

Risk of Bias
- (+) Low
- (−) Some
- (×) High
